# MSIseq: Software for Assessing Microsatellite Instability from Catalogs of Somatic Mutations

**DOI:** 10.1038/srep13321

**Published:** 2015-08-26

**Authors:** Mi Ni Huang, John R. McPherson, Ioana Cutcutache, Bin Tean Teh, Patrick Tan, Steven G. Rozen

**Affiliations:** 1 Centre for Computational Biology, Duke-NUS Graduate Medical School, Singapore, 169857, Singapore; 2 Cancer and Stem Cell Biology Program, Duke-NUS Graduate Medical School, Singapore, 169857, Singapore; 3 Laboratory of Cancer Epigenome, Division of Medical Sciences, National Cancer Centre Singapore, Singapore, 169610, Singapore; 4 Cancer Science Institute of Singapore, Yong Loo Lin School of Medicine, National University of Singapore, Singapore, 119074, Singapore; 5 Division of Cellular and Molecular Research, National Cancer Centre Singapore, Singapore, 169610, Singapore; 6 Genome Institute of Singapore, A*STAR, Singapore, 138672, Singapore

## Abstract

Microsatellite instability (MSI) is a form of hypermutation that occurs in some tumors due to defects in cellular DNA mismatch repair. MSI is characterized by frequent somatic mutations (i.e., cancer-specific mutations) that change the length of simple repeats (e.g., AAAAA…., GATAGATAGATA...). Clinical MSI tests evaluate the lengths of a handful of simple repeat sites, while next-generation sequencing can assay many more sites and offers a much more complete view of their somatic mutation frequencies. Using somatic mutation data from the exomes of a 361-tumor training set, we developed classifiers to determine MSI status based on four machine-learning frameworks. All frameworks had high accuracy, and after choosing one we determined that it had >98% concordance with clinical tests in a separate 163-tumor test set. Furthermore, this classifier retained high concordance even when classifying tumors based on subsets of whole-exome data. We have released a CRAN R package, MSIseq, based on this classifier. MSIseq is faster and simpler to use than software that requires large files of aligned sequenced reads. MSIseq will be useful for genomic studies in which clinical MSI test results are unavailable and for detecting possible misclassifications by clinical tests.

Microsatellite instability (MSI) is a form of hypermutation caused by defective DNA mismatch repair (MMR). MSI is characterized by widespread changes in the length of genomic mononucleotide repeats (e.g., AAAAA….) or microsatellites (e.g., GATAGATAGATA….), collectively termed simple repeats^1^,^2^,^3^. MSI is also characterized by high rates of single-nucleotide-substitution (SNS) mutations^4^. MSI can arise due to germ-line mutations in MMR genes, due to somatic mutations in MMR genes, or due to epigenetic inactivation of MMR genes^5^,^6^.

MSI was first reported in colorectal cancer in 1993, and it proved to be a marker of favorable prognosis^7^,^8^,^9^,^10^,^11^. Some individuals have heterozygous germ-line defects in an MMR gene and consequently develop cancers at young ages due to subsequent inactivation of the functional homolog. Clinical MSI testing to diagnose this condition, known as Lynch syndrome, is well established^12^,^13^.

MSI is assessed by measuring the lengths of a set of mono- and/or dinucleotide repeats in tumor and matched normal DNA. Several DNA-based clinical tests for MSI are in widespread use. The Bethesda panel consists of two mono- and three dinucleotide repeats^2^. The Promega panel consists of the two mononucleotide repeats used in the Bethesda panel plus three additional mononucleotide repeats^14^. This panel also uses two pentanucleotide repeats to check for tumor mix-ups or contamination. The MSI-Mono-Dinucleotide Assay used by the Cancer Genome Atlas (TCGA) consists of the Bethesda panel plus two additional mononucleotide repeats^15^,^16^,^17^. In addition, some laboratories use different or extended panels of repeat markers^18^. Tumors in which ≥40% of the markers in a panel show somatic length mutations are generally termed MSI-high (MSI-H)^19^. Tumors in which no markers show length mutations are termed microsatellite stable (MSS). The remaining tumors are sometimes termed MSI-low (MSI-L). As discussed below, for several reasons, MSI-L tumors are often grouped with MSS tumors.

With the emergence of next-generation sequencing (NGS) technologies, tumors can be sequenced quickly and cheaply for research and, sometimes, for personalized cancer treatment^20^,^21^,^22^. However, MSI testing is not routine in many clinical situations, and only limited clinical information is available for much published tumor-sequence data. We also note that NGS exome data cannot directly reveal mutations at the simple repeat sites used in laboratory tests, because these sites are non-exonic. Thus, a method to determine MSI status from NGS data alone, and in particular from whole-exome data or data from targeted subsets of the exome, would be very useful, especially because MSI has significant implications for tumor etiology and biology and for prognosis. Furthermore, when exome-based somatic mutation data are generated, a robust prediction could also obviate the need for a conventional clinical MSI assessment.

A literature search reveals only two published programs, MSIsensor^23^ and mSINGS^24^, for determining MSI status from NGS data, both of which operate on “BAM” files, the files that contain aligned reads and their base- and mapping-quality scores. In addition, there is a method that operates on RNA-seq BAM files to determine MSI status, although no software implementing this method has been released^25^. Given that pipelines for analyzing matched tumor and normal genome sequence data typically generate lists of somatic single nucleotide mutations and micro insertions and deletions, including those at mononucleotide and microsatellite repeats, it would be simpler and desirable to determine MSI status from these lists. Thus, our aims are to: (1) generate robust software capable of reliably determining MSI status from lists of somatic mutation calls, (2) evaluate the accuracy of this software on a test set independent of the training set on which it was developed, and (3) release this software under an open source license.

## Methods

### Sources of somatic mutation lists and MSI statuses for exomes

We obtained publicly available data on somatic mutations from whole-exome sequencing and on laboratory-determined MSI statuses for 526 whole exomes as follows.

For gastric adenocarcinoma, we used whole-exome somatic mutation data from reference^26^ (14 tumors) and reference^18^ (22 tumors). For the tumors from reference^26^, MSI statuses had been determined by the Promega MSI Analysis System Version 1.2 (Promega Corp, USA)^14^. For the tumors from reference^18^, MSI statuses had been determined by an extended panel of markers as described. From the two references, we called somatic mutations in these tumors using the Genome Analysis Toolkit (GATK) (https://www.broadinstitute.org/gatk/) pipeline described in reference 27.

For colon (216 tumors), rectal (81 tumors) and endometrial (193 tumors) carcinomas, we obtained somatic mutation data^16^,^17^ from the TCGA data portal (https://tcga-data.nci.nih.gov/tcga/) on 3 July 2013. At the TCGA data portal, we chose the specific cancer type (e.g. colon adenocarcinoma) and then chose the publicly available Mutation Annotation Files (MAFs) for level 2 exome data and clicked “Build Archive”. The data portal then prepared the data and sent a link for download. Because it would be difficult for other researchers to download the MAFs as they were on the date we downloaded them, we will make the specific MAFs we used available on request. The MSI-Mono-Dinucleotide Assay^15^ had been used to determine the MSI statuses of these tumors. Somatic mutations in the colon and rectal tumors were called by Baylor College of Medicine using GATK and Atlas2 (https://www.hgsc.bcm.edu/software/software/atlas-2). Somatic mutations in the endometrial tumors were called by the Broad Institute using GATK.

### Sources of somatic mutation lists for whole genomes

We obtained published whole-genome somatic mutation data and MSI statuses from 100 tumors reported in reference 28. Somatic mutations from the exomes of three of these 100 tumors were reported in reference 18 and were used in our whole exome analysis. The whole-genome somatic mutations in reference 28 were identified by Strelka^29^.

### Sources of BAM files

In order to compare run times and prediction accuracy of our methods with those of other methods that operate on BAM files, we obtained the exome BAM files of 22 gastric tumor-normal pairs from reference^18^. We also obtained genome BAM files of 2 genome gastric tumor-normal pairs from reference 30. The MSI statuses of the 2 whole-genome sequenced tumors were determined by Promega MSI Analysis System Version 1.2 (Promega Corp, USA)^14^.

### Classification categories

**Figure 1:**
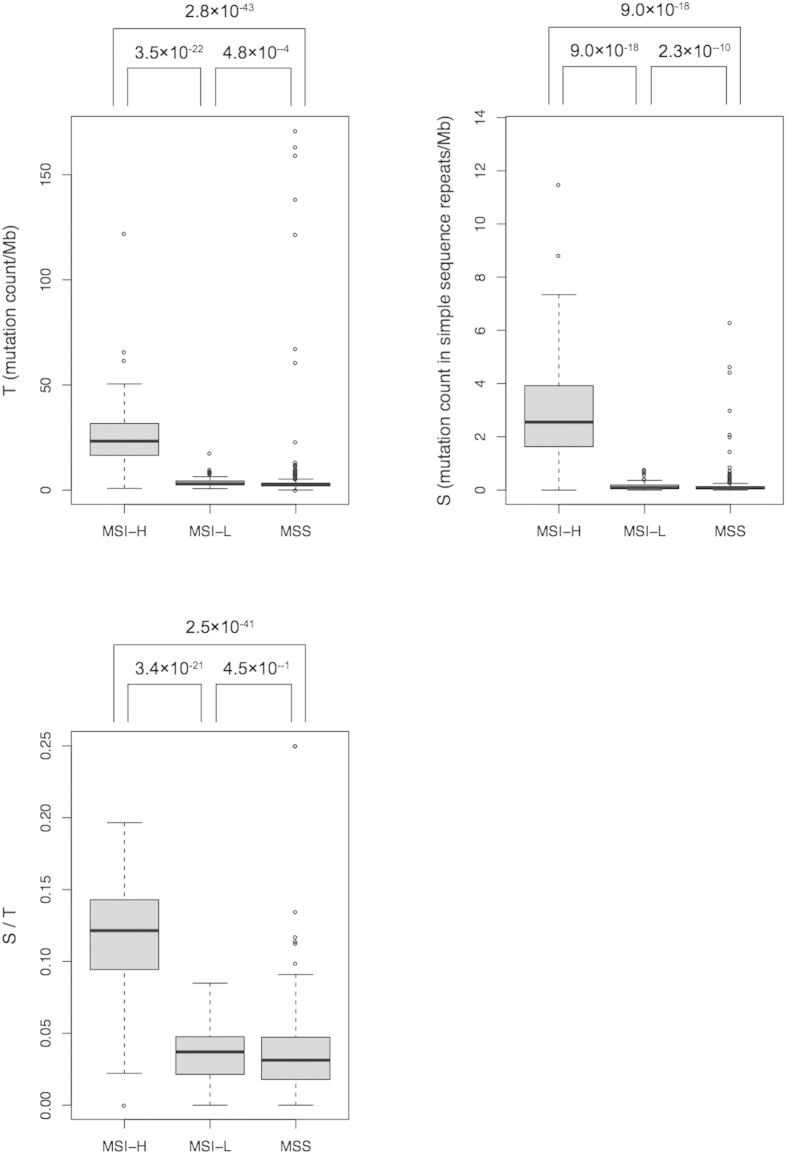
Variation in *T*, *S*, and *S*/*T* across TCGA’s three laboratory-based MSI categories: MSI-H, microsatellite instable high; MSI-L, microsatellite instable low; MSS, microsatellite stable. P values by Wilcoxon rank-sum tests. Dark horizontal lines are medians; boxes extend from first to third quartiles; whiskers mark the most extreme data points that are ≤1.5 times the length of the box distant from the box.

For classification, we grouped MSI-L and MSS tumors together as “non-MSI-H” for the following reasons. First, although the TCGA tumors were assigned to one of three MSI classes, MSI-H, MSI-L and MSS, in terms of clinical significance MSS and MSI-L tumors are similar to each other but different from MSI-H tumors^2^,^31^,^32^,^33^. Furthermore, MSS and MSI-L tumors are very similar in terms of: (i) total somatic mutation count (both microindels—small insertions or deletions—and SNSs) per megabase (Mb) (*T*), (ii) mutation count per Mb in simple repeats (*S*), and (iii) *S*/*T* (Fig. 1). There were large and significant differences in *T*, *S*, and *S*/*T* between the MSI-L and MSI-H tumors and between MSS and MSI-H tumors, but not between the MSI-L and MSS tumors (Fig. 1). The data for the gastric tumors categorized them only as MSI-H and non-MSI-H^18^,^26^. The proportions of MSI-H tumors were as follows: colon, 40/216, rectal, 3/81, endometrial, 54/193, gastric, 5/36.

### Developing the classifier

We use the somatic mutation data in the 526 exome-sequenced tumors for developing and testing our classifier.

For each tumor, we required a catalog of somatic SNSs and microindels. Because MMR deficiency leading to MSI likely affects rates of SNS and microindel mutations differently and because variant calling for microindels is less reliable than for SNSs, we considered these two types of mutations separately. MMR deficiency leads to notably high microindel rates in simple repeats; it is these high rates that gave rise to the term microsatellite instability. Therefore, we required an annotation of the exome indicating the locations of simple repeats, including both mononucleotides and microsatellites. We considered mononucleotides of length ≥5, as annotated by a function provided in the MSIseq package (described below, http://cran.r-project.org/web/packages/MSIseq/index.html) that examined GRCh37 (Genome Reference Consortium Human Reference 37). We considered microsatellites consisting of di-, tri-, and tetranucleotide repeats, as annotated in the “simpleRepeats” table from UCSC genome annotation database (http://hgdownload.cse.ucsc.edu/goldenpath/hg19/database/ downloaded March, 2013). We also took into account the total length of the sequence that was targeted for hybridization capture and sequencing. We used this as the denominator to obtain mutation counts per Mb. In addition to information on mutations, we also included the type of cancer as a possible input variable, because it might conceivably affect the mutation signature of MSI.

We used the following variables as candidate inputs to the classifiers that we tested:

*T.sns*, number of SNSs in all sequences/Mb

*S.sns*, number of SNSs in simple sequence repeats/Mb

*T.ind*, number of microindels in all sequences/Mb

*S.ind*, number of microindels in simple sequence repeats/Mb

*T*, number of mutations (SNSs and microindels) in all sequences/Mb

*S*, number of mutations (SNSs and microindels) in simple sequence repeats/Mb

*S.sns*/*T.sns*

*S.ind*/*T.ind*

*S*/*T*

Cancer type (colon, rectal, endometrial, or gastric)

We used the R function sample() across 526 tumors to randomly select a training set of 363 tumors and a test set of 163 tumors. Because all 5 gastric MSI-H tumors were assigned to the training set, for better distribution we randomly reassigned 2 gastric MSI-H tumors to the test set. The final training set contained 361 tumors, and the test set contained 165 tumors.

We evaluated the following machine learning frameworks provided by the R package RWeka (http://cran.r-project.org/web/packages/RWeka/, version 3.7.2)^34^,^35^: logistic regression [function Logistic()], decision tree [function J48()], random forest [make_Weka_classifier("weka/classifiers/trees/RandomForest")], and naïve Bayes [make_Weka_classifier("weka/classifiers/bayes/NaiveBayes")]. We carried out five-fold cross validation in the training set using the function evaluate_Weka_classifier(). The R package MSIseq (presented below) provides details of the software that we developed based on RWeka. MSIseq is available at The Comprehensive R Archive Network (CRAN), http://cran.r-project.org/web/packages/MSIseq/index.html under the standard GPL3 open-source license.

## Results

### Classifier selection and evaluation

When initially trained on the full variable set as described above, the four machine learning frameworks (logistic regression, decision tree, random forest, and naïve Bayes) performed similarly and had high cross validation concordance with laboratory-determined MSI status (ranging from 96.5% to 98.6%, Table 1). This suggested that the input variables contained adequate information for MSI classification. Among the frameworks, the decision tree exhibited the highest concordance with laboratory tests (Table 1%). We chose this classifier for further investigation both because of its high concordance and because of ease of interpretation, as it used only one variable, *S.ind*. Only this variable out of the possible 10 variables was used, because RWeka simplified the decision trees based on standard principles^36^.

We term this decision-tree classifier NGSclassifier. In the test set, NGSclassifier's concordance with laboratory-assessed MSI status was 98.8%; thus, the classifier performed well on the test set as well as the training set. As noted, NGSclassifier depends only on *S.ind,* which is the number of microindels in simple sequence repeats per Mb, i.e. the number of somatic length-change mutations in simple repeats per Mb; tumors with *S.ind* > 0.395 are classified as MSI-H. This is a plausible criterion that reflects the biological concept of instability in the lengths of simple repeats.

**Figure 2:**
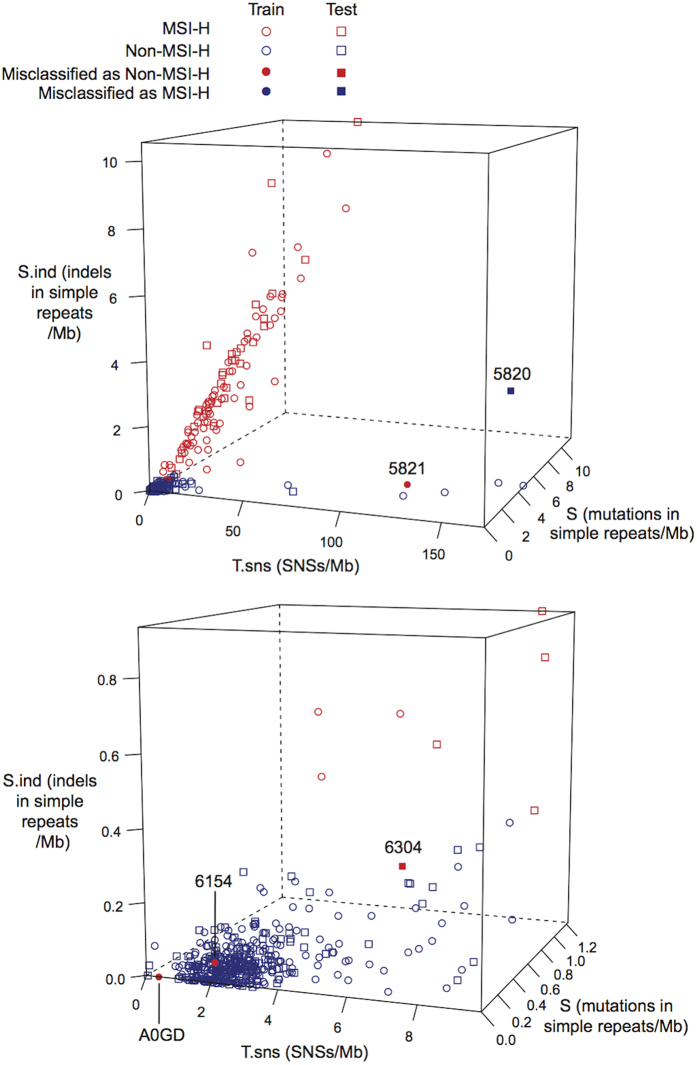
3-D plot of the variables *S.ind*, *T.sns*, and *S* in the training and test sets. The lower panel is a close-up view for *S.ind*  ≤1, *T.sns*  ≤10, and *S*  ≤1.23. Tumors with discordant classification by NGSclassifier and laboratory tests are labeled by the last four characters of the tumor identifier.

Figure 2 plots the tumors according to *T.sns*, *S.ind* and *S*. Most MSI-H tumors had high *S.ind* and high *T.sns*, shown as a cloud of red points extending up and to the right in the top panel of Fig. 2. Among these tumors, *T.sns* and *S* tend to grow linearly with *S.ind,* which is consistent with the fact that deficiency of DNA mismatch repair functionality leads not only to frequent length changes of simple repeats but also to higher SNS rates over the entire genome (*T.sns*) and higher overall mutation rates in simple repeats (*S*)^4^.

Figure 2 also shows 8 tumors with *T.sns* > 60, but with relatively low levels of *S.ind*. Of these, 6 were classified as non-MSI by both NGSclassifier and laboratory tests (Table 2). The somatic trinucleotide mutation spectra of these tumors showed high frequencies of TCT >TAT and TCG >TTG substitutions (Supplementary Figure 1). These substitutions, combined with very high *T.sns*, are characteristic of tumors with mutations in the exonucleolytic proofreading domain of the gene *POLE* [polymerase (DNA directed), epsilon, catalytic subunit]^37^. Five of the 6 tumors with *POLE*-associated mutation signatures had non-silent somatic mutations in *POLE* (Table 2). This was a much higher proportion than in tumors without the *POLE*-associated signature (p = 9.4 ×10^−7^, Table 3, Fisher’s exact test, one-sided).

The other two hypermutated tumors were discordantly classified (Table 2, Fig. 2, top panel). These tumors showed very few of the TCT >TAT or TCG >TTG substitutions associated with *POLE* mutations. Instead they had extremely high proportions of CG >TG mutations and somewhat high proportions of usually-rare T>C mutations (Supplementary Figure 1). Both tumors have *S.ind* values less than many tumors that are MSI-H, but have *T.sns* higher than all MSI-H tumors. These tumors may reflect an unknown hypermutagenic process.

In addition, three other tumors categorized as MSI-H by laboratory tests were classified discordantly as non-MSI-H by NGSclassifier because they had *S.ind* < 0.395. Two of these tumors also had low values of *T.sns* and *S*, suggesting the possibility that they in fact had intact MMR functionality. Consistent with this possibility, neither of these two tumors had a non-silent mutation in an MMR gene. The third tumor (TCGA-G4-6304, Table 2 and Fig. 2, lower panel) had *S.ind* below but close to the cutoff of 0.395 and relatively high *T.sns* and *S*. This tumor could be a boundary case in which, for example, MMR deficiency might have arisen only late in tumor development, resulting in relatively few simple-repeat-length changes and relatively few SNSs.

### Robustness of NGSclassifier on somatic mutations from subsets of the exome and from the whole genome

**Figure 3:**
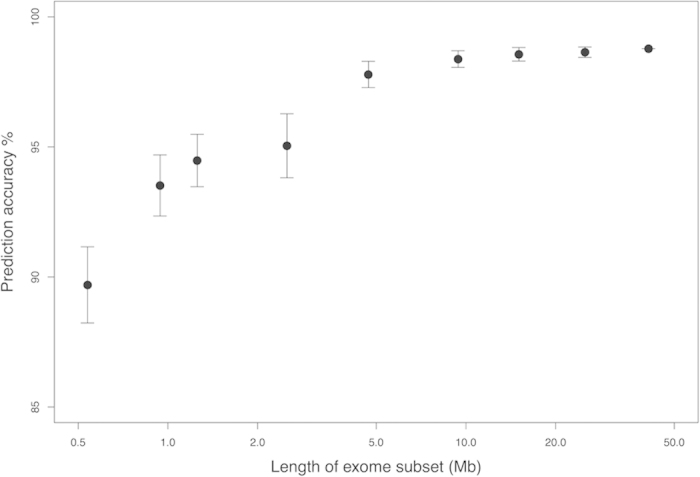
Prediction accuracy of NGSclassifier (*y* axis) on exome subsets of varying lengths (*x* axis). “Length of exome subset" on the *x* axis refers to the region that was targeted for sequencing. The prediction accuracy is the number of tumors with concordant MSI status between NGSclassifier and the laboratory test, divided by the total number of tumors. Error bars indicate standard deviations for 1,000 different, random exome subsets at each length. Supplementary Table 1 shows the underlying data.

To test NGSclassifier's performance on panels of selected genes comprising less sequence than an exome, we applied NGSclassifier to random subsets of whole-exome sequencing target regions with total lengths varying from 0.54 to 25 Mb (Fig. 3). NGSclassifier was robust even when the length of the exome subset was only 4.7 Mb, at which length NGSclassifier’s average accuracy was >98%. The density of simple repeats as defined in Methods is not entirely uniform. The average is 5.6/Kb and the standard deviation is 0.7/Kb in exome-sequencing target regions, based on consecutive groups of 1,000 target regions (usually exons). Random resampling of smaller subsets of the exome, as in Fig. 3, reduces this variation, which in this situation does not impede use of NGSclassifier. However, for regionally localized subsets of the exome, it might be necessary to retrain the classifier to account for regional differences in simple-repeat density.

We also tested NGSclassifier on somatic mutations from 100 whole-genome-sequenced tumors. Because the frequency of simple repeats across the genome (7.4/Kb) is higher than in the exome (5.6/Kb), we trained a new classifier using the same decision tree framework on a training set of 60 tumors (randomly selected from the 100 tumors). This classifier achieved 100% prediction accuracy in the test set of 40 tumors. The new classifier had a cut off as *S.ind* < 0.909.

### Minimum sequencing depth requirement

**Figure 4:**
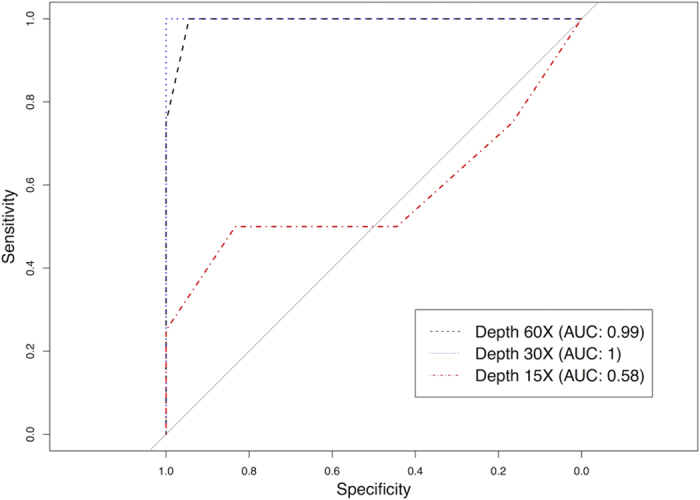
30× depth provides adequate somatic variant calls for NGSclassifier. Shown are MSI-status classification receiver operating characteristic (ROC) curves. *S.ind* was calculated from the mutations list generated by a GATK pipeline similar that used in reference 18. Full-depth or down-sampled exome BAM files from 22 tumor-normal pairs were analyzed. AUC, area under the curve.

Sequencing depth affects somatic mutation calling, which in turn would presumably affect the performance of NGSclassifier. To assess the influence of sequencing depth on NGSclassifier's performance, we randomly down-sampled the reads of the BAM files from 22 tumor-normal pairs from reference^18^, and called variants on the down-sampled BAM files using the GATK pipeline similar to the one used in this reference. We calculated NGSclassifier's receiver operating characteristic (ROC) curves on these variant calls (Fig. 4). We found that 30× depth, which is usually considered too low for tumor-normal sequencing, nevertheless provided an area under the curve of 1.0. However, 15× depth was clearly insufficient.

### R package implementing NGSclassifier

**Figure 5:**
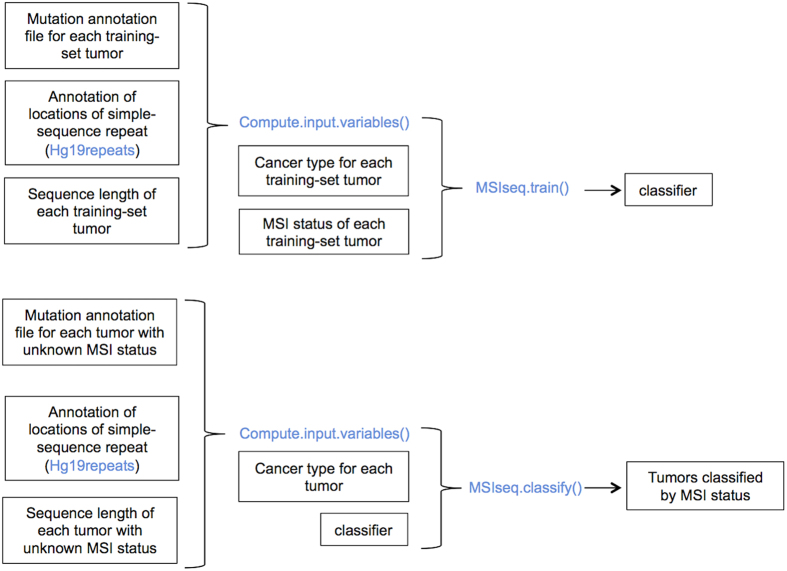
Workflow for the R MSIseq package. Functions and variables in the package are highlighted in blue. MSIseq provides Compute.input.variables() to calculate the potential input variables (*S.ind*, *T.sns*, etc.) from (i) a mutation annotation file, (ii) an annotation of the locations of simple repeats in the genome, and (iii) the lengths of the sequenced regions of the genome that were searched for somatic mutations. MSIseq provides these data as used in this paper in the variables NGStraindata, Hg19repeats, and NGStrainseqLen. MSIseq.train() takes the input variables plus (optionally) cancer type information and creates a classifier. Please refer to the MSISeq documentation and vignette for details. MSIseq also provides a pre-computed classifier (called NGSclassifier in the package) that implements the NGSclassifier presented in this paper. For classification of samples with unknown MSI status, input variables can be prepared from the mutation annotation file by Compute.input.variables() and then passed to MSIseq.classify() along with a classifier generated by MSIseq.train().

We have created an R package, MSIseq, that implements NGSclassifier and is available at CRAN (http://cran.r-project.org/web/packages/MSIseq/index.html)^38^. In addition to NGSclassifier, MSIseq also provides the ability to retrain the classifier (Fig. 5). MSIseq provides two main functions, MSIseq.train() and MSIseq.classify(). The first function, MSIseq.train(), generates a classifier from training data. The second function classifies tumors using classifiers generated by MSIseq.train(). The ability to train a new classifier [provided by function MSIseq.train()] is important for future use of MSIseq for two reasons. First, variant calling methods may improve, especially with respect to microindels, and this may necessitate re-tuning the tree model. Second, with inclusion of additional cancer types in the model (for example, esophageal cancer, for which no training data were available) it may be necessary to include cancer type as an input variable. MSIseq also provides a helper function, Compute.input.variables (), to generate the input variables (*T.sns*, *S.sns*, *T.ind*, etc.) needed by these two functions given (1) Mutation Annotation Files (“MAF files”) that provide the locations of somatic mutations from a collection of tumors and (2) a file containing the genomic locations of simple repeats in the genome. Training, including 5-fold cross validation, on 361 tumors required 183 seconds elapsed time on a Mac with a 2.9 GHz Intel I7 core and 8 gigabytes of random access memory. Classification of all of the tumors in the test set required 162 seconds elapsed time.

As noted above, POLE-deficient tumors showed very different characteristics compared to MSI tumors, and MSIseq is able to identify possible POLE-deficient tumors. However, since extensive training data are not available (only 6 out of 526 exomes were from POLE-deficient tumors), MSIseq simply flags samples with *T.sns* > 60/Mb and *S.ind* < 0.18/Mb as possible POLE-deficient tumors.

## Discussion

We have described an in-silico MSI-status classifier, NGSclassifier, that is available in the R package MSIseq and that operates on somatic mutation data extracted from NGS of whole exomes or subsets of the exome as short as 4.7 Mb. NGSclassifier’s accuracy was 98.6% in a whole-exome-based training set and 98.78% in a test set. The high concordance of this classifier with laboratory tests and the high concordance of multiple machine-learning frameworks (Table 1) indicate that catalogs of somatic mutations from whole-exome NGS contain sufficient information for assessing MSI status, and by extension, underlying deficiencies in MMR. Two of the discrepancies between NGSclassifier and laboratory tests were due to tumors that laboratory tests categorized as MSI-H even though they had very few somatic length changes in simple repeats (*S.ind*) and very few somatic SNSs, suggesting that they may have had intact MMR activity. A third tumor with discrepant MSI status may have been a boundary case. The two other tumors with discrepant MSI-status may represent unknown hypermutational processes.

A literature and web search revealed only two software packages, MSIsensor^23^ and mSINGS^24^ that determine MSI status from NGS data, both of which, unlike MSIseq's NGSclassifier, operate on the aligned reads in complete (and often very large) BAM files rather than on lists of somatic mutations. MSIsensor examines reads in matched tumor and normal BAM files to calculate a score consisting of the percentage of simple-repeat sites in the exome that show evidence of MSI. Although it used all the read data from mononucleotide repeats and microsatellites in the BAM files, MSIsensor's accuracy, based on training set data, was 99% (1 out of 71 MSI-H tumors and 1 out of 268 non-MSI-H tumors discordantly categorized). This estimate of MSIsensor’s accuracy is likely to be somewhat optimistic, as it based on training set data; MSIsensor's discordance rate of 2/239 in the training set is practically and statistically indistinguishable from NGSclassifier’s rate in the test set (2/165). Results from MSIsensor have been reported only for exome data, but not for targeted panels of small subsets of the exome.

Like MSIsensor, mSINGS examines reads in BAM files, but unlike NGSclassifier or MSIsensor, mSINGS examines only the BAM file from the tumor; data from a matched normal sample is not needed. mSINGS achieved 100% accuracy in a training set of 12 exome-sequenced tumors and 96% accuracy in a training set of 28 tumors subjected to sequencing to a targeted panel 234 genes. These values are likely to be somewhat optimistic, as they are based on training set data; they are statistically indistinguishable from NGSclassifier's accuracy in the test set.

Unlike MSIsensor, and mSINGS, which operate on large BAM files, MSIseq operates on the much smaller lists of somatic variants that are generated by most pipelines for identifying these mutations in next-generation sequencing data from tumor-normal pairs. To assess the relative computational time needed by each of MSIsensor, mSINGS, and MSIseq, we tested them on exome BAM files from 22 gastric tumor-normal pairs or, in the case of MSIseq, on somatic mutations called from these BAM files. We tested the programs on a Linux computer with an Intel® Xeon® CPU E5420 running at 2.50 GHz. MSIseq was on average 90 times faster than MSIsensor and 79 times faster than mSINGS (Table 4). We also tested the programs on whole-genome BAM files from 2 gastric tumor-normal pairs, or, for MSIseq, on somatic mutation calls from these BAM files. MSIsensor exited with a segmentation fault. mSINGS failed due to inadequate sequencing depth (i.e. depth < 30×) in a specific group of mononucleotide sites that mSINGS must assess.

In conclusion, we have released a robust, reusable R package, MSIseq, that implements NGSclassifier and that can also train a new classifier based on the same framework. For genomic studies based on NGS whole-genome, whole-exome data or on data from targeted subsets of the exome, MSIseq will be useful when laboratory tests of MSI status are not available and for detecting possible miscategorizations by laboratory tests.

## Additional Information

**How to cite this article**: Ni Huang, M. *et al.* MSIseq: Software for Assessing Microsatellite Instability from Catalogs of Somatic Mutations. *Sci. Rep.* **5**, 13321; doi: 10.1038/srep13321 (2015).

## Supplementary Material

Supplementary Information

## Figures and Tables

**Table 1:** For each of four machine-learning frameworks, percent of training-set tumors with predicted MSI status concordant with laboratory tests in five-fold cross validation.

	Machine learning framework			
	Logisticregression	Decisiontree	Randomforest	NaïveBayes
Percent concordant	96.5	98.6	98.1	96.7

**Table 2:** Hypermutated tumors (*T.sns* > 60/Mb) and tumors with discordant MSI status between NGSclassifier and laboratory tests.

Sample ID	Training set?	*S.ind*	MSI-H by laboratory test?	MSI-H by MSIseq?	*POLE*-like Mutation signature?	Mutation in *POLE*^a^
Hypermutated tumors with concordant MSI status						
TCGA-F5-6814	Y	0.045	N	N	Y	None
TCGA-CA-6717	Y	0.11	N	N	Y	Exo
TCGA-AZ-4315	Y	0.045	N	N	Y	Other
TCGA-EI-6917	Y	0.091	N	N	Y	Exo
TCGA-AA-3510	N	0.023	N	N	Y	Other
TCGA-CA-6718	Y	0.023	N	N	Y	Exo
Hypermutated tumors with discordant MSI status						
TCGA-AM-5821	Y	0.25	Y	N	N	None
TCGA-AM-5820	N	2.61	N	Y	N	None
Other tumors with discordant MSI status						
TCGA-A5-A0GD	Y	0.00	Y	N	N	None
TCGA-DC-6154	Y	0.045	Y	N	N	None
TCGA-G4-6304	N	0.27	Y	N	N	None

*S.ind*: number of microindels in simple repeats/Mb.

*POLE*: the polymerase (DNA directed), epsilon, catalytic subunit gene.

^a^Exo, a non-silent mutation in the exonuclease domain of *POLE*; Other, a non-silent mutation in another domain of *POLE*

**Table 3:** Fisher test for non-silent mutations in the *POLE* gene. “*POLE* signature” refers to very high rates of TCT > TAT and TCG > TTG mutations (Supplementary Figure 1).

		*POLE* signature?	
		Y	N
Non-silent mutation in *POLE*?	**Y**	5	20
	**N**	1	500

**Table 4:** CPU times and prediction accuracy for analyzing 22 gastric cancer tumor-normal exomes for MSIseq, MSIsensor and mSINGS.

	MSIseq	MSIsensor	mSINGS
Average CPU time per tumor-normal pair (min)	0.28	22.35	25.45
MSI status classification accuracy (%)	100	100	100
