# Supplementary material for: MSIseq: Software for Assessing Microsatellite Instability from Catalogs of Somatic Mutations
Source: Sci Rep. 2015 Aug 26;5:13321. doi: 10.1038/srep13321 (PMC4549793; doi:10.1038/srep13321)

Supplementary Information for Huang et al. "MSIseq: Software for Assessing Microsatellite Instability from Catalogs of Somatic Mutations"

Mi Ni Huang[1,2], John R. McPherson[1,2], Ioana Cutcutache[1,2], Bin Tean Teh[2,3] Patrick Tan[2,4,5,6] and Steven G. Rozen[1,2,*]

[1]Centre for Computational Biology, Duke-NUS Graduate Medical School, Singapore, 169857, Singapore, [2]Cancer and Stem Cell Biology Program, Duke-NUS Graduate Medical School, Singapore, 169857, Singapore, [3]Laboratory of Cancer Epigenome, Division of Medical Sciences, National Cancer Centre Singapore, Singapore, 169610, Singapore, [4]Cancer Science Institute of Singapore, Yong Loo Lin School of Medicine, National University of Singapore, Singapore, 119074, Singapore, [5]Division of Cellular and Molecular Research, National Cancer Centre Singapore, Singapore, 169610, Singapore, [6]Genome Institute of Singapore, A*STAR, Singapore, 138672, Singapore

* To whom correspondence should be addressed.

Contents

Supplementary Table 1. NGSclassifier accuracy on somatic mutations from sequences shorter than an exome. 3

Supplementary Figure 1. Trinucleotide mutation spectra of hypermutated tumors. 4

Supplementary Table 1. NGSclassifier accuracy on somatic mutations from sequences shorter than an exome.
Shown are the average accuracy and standard deviation of 1,000 random exome subsets comprising sequence of each of the indicated lengths. Accuracy was assessed on the training and test sets combined. These data are plotted in Figure 3 in the main text.

	**Sequenced length (Mb)**								
	**41 (whole exome)**	**25**	**15**	**9.4**	**4.7**	**2.5**	**1.3**	**0.94**	**0.54**
**Average accuracy (%)**	98.78	98.64	98.56	98.38	97.79	95.04	94.48	93.52	89.70
**Standard deviation**	N/A	0.199	0.260	0.319	0.506	1.232	1.007	1.175	1.464

Supplementary Figure 1. Trinucleotide mutation spectra of hypermutated tumors. The first 6 tumors show strong signatures of mutations in the gene *POLE* [polymerase (DNA directed), epsilon, catalytic subunit] (TCT > TAT and TCG > TTG). The last two are hypermutated but show no evidence of the *POLE* signature. See also Table 2 in the main text.

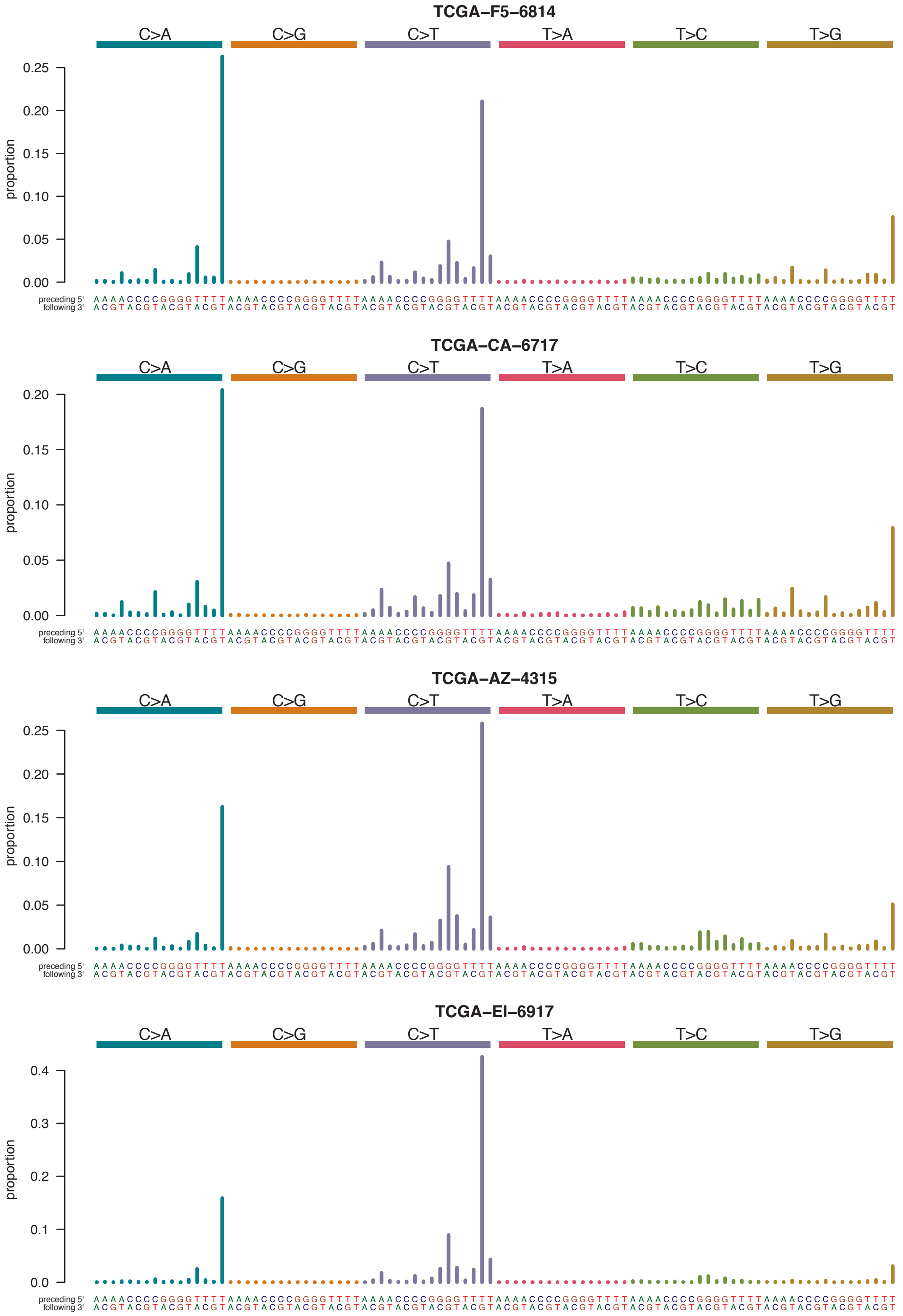

(Supplementary Figure 1, continued)

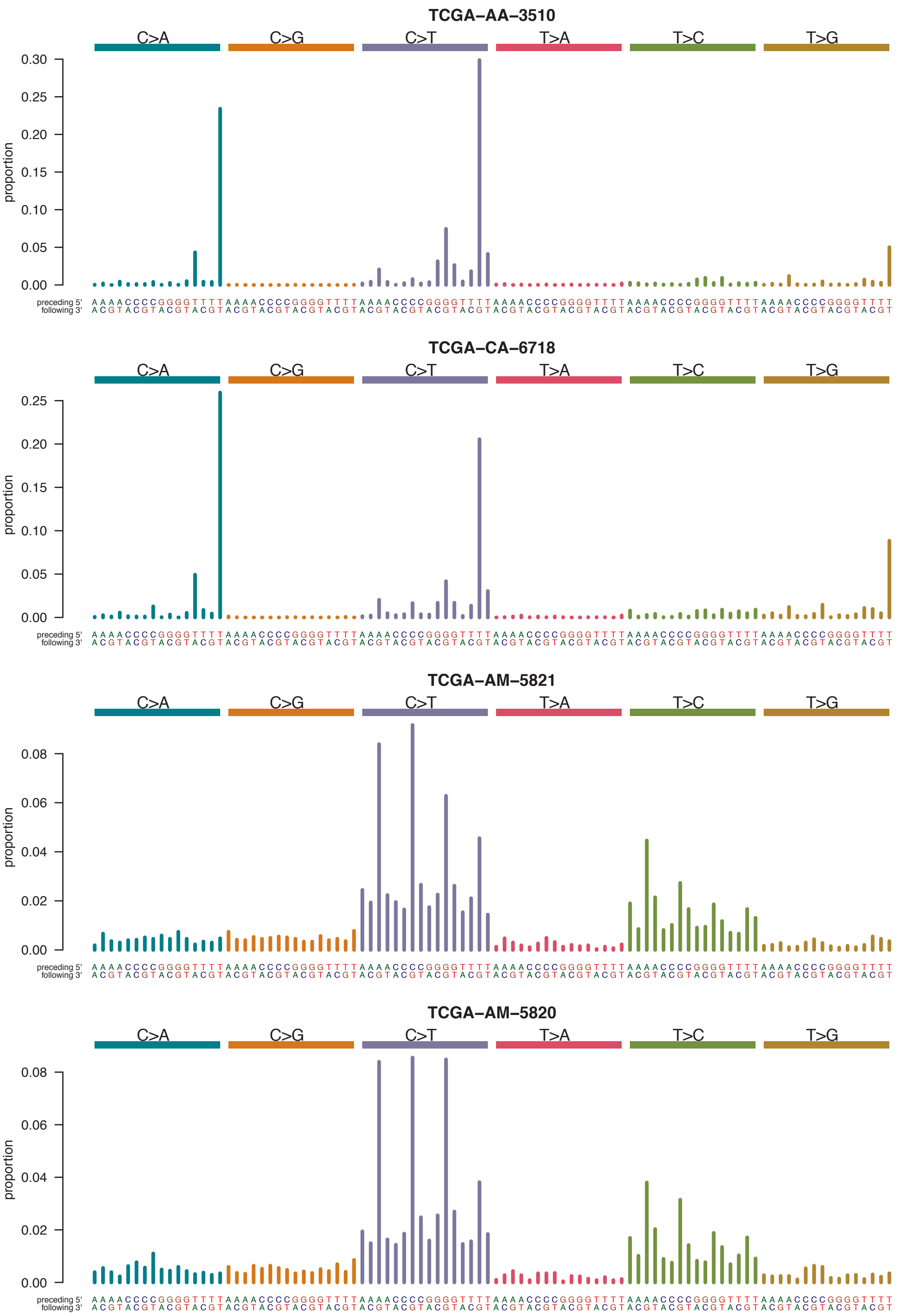

Supplement: Supplementary Information [file srep13321-s1.pdf]
